# Assessment of Parental Needs and Quality of Life in Children with a Rare Neuromuscular Disease (Pompe Disease): A Quantitative–Qualitative Study

**DOI:** 10.3390/bs13120956

**Published:** 2023-11-21

**Authors:** Loredana Benedetto, Olimpia Musumeci, Annunziata Giordano, Mattia Porcino, Massimo Ingrassia

**Affiliations:** 1 Department of Clinical and Experimental Medicine, University of Messina, AOU “G. Martino”, 98125 Messina, Italy; loredana.benedetto@unime.it (L.B.); olimpia.musumeci@unime.it (O.M.); nunzia44@live.it (A.G.); mattia.porcino@studenti.unime.it (M.P.); 2 Unit of Neurology and Neuromuscular Disorders, Regional Reference Centre for Rare Neurological and Neuromuscular Diseases, AOU “G. Martino”, 98125 Messina, Italy

**Keywords:** childhood rare diseases, Pompe disease, quality of life, parental needs, prevention of burden

## Abstract

Pompe disease (PD) is a rare metabolic disorder with progressive neuromuscular consequences that negatively impact a child’s development and quality of life (QoL). Despite an improved prognosis with treatment, the risk for early death due cardiorespiratory crisis remains. Parents not only face physical fatigue and family distress in coping with the child’s special needs but also experience emotions, worries, and unexpressed needs (a “humanistic burden”) that require supportive interventions. Fourteen parents of children with PD completed an online self-report questionnaire assessing their child’s QoL, their own parental burden of care, and disease-related issues. The aim was to estimate the associations between the child’s QoL and the caregiver’s burden levels. Three mothers were also interviewed. A total of 57.1% of parents lived with moderate/severe burden conditions; worse QoL for the child was associated with higher levels of caregiver burden (*r*_S_[*N* = 14] = −0.67, *p* < 0.01). Uncertainty about the child’s future was a state commonly described by mothers. However, the child’s resilience, normalization of disease, and coping strategies (primarily positive appraisal and focusing on the present) alleviated suffering and helped mothers maintain family functioning. Finally, dissatisfaction with communication in relationships with professionals emerged. In conclusion, a typical pediatric palliative care approach is recommended since it manages to guarantee parents empathetic and supportive communication from healthcare professionals, alleviating feelings of isolation and loneliness in parents.

## 1. Introduction

Rare diseases are defined by their low prevalence within the population, yet their psychological impact is devastating. Pompe disease (PD or glycogen storage disease type II, GSD II) is a rare inherited metabolic disorder characterized by a deficiency of the enzyme acid alpha-glucosidase (GAA), which induces an accumulation of glycogen mainly in skeletal muscle, leading to slowly progressive muscle weakness, resulting in walking disability and reduced respiratory function.

Based on the age of onset, residual GAA activity, and clinical phenotype, we can distinguish two different clinical entities [1,2]. The first is infantile-onset Pompe disease (IOPD), characterized by an early onset within the first year of life, a rapid progression, and a more severe prognosis. The second is late-onset Pompe disease (LOPD), which can manifest from childhood to adulthood and presents with a lower progression and less severe outcomes. Generally, children with IODP present cardiomyopathy, heart failure, marked hypotonia, and respiratory failure, leading if untreated to a grim prognosis due to cardiorespiratory complications [3]. The current treatment for PD at all ages is enzyme replacement therapy (ERT), which reduces the risk of early death [4,5]. Early identification through newborn screening (NBS) is essential for early treatment and to improve the prognosis [6,7]. 

### 1.1. Health-Related Quality of Life in Infantile PD

Respiratory distress and feeding difficulties are commonly observed in infantile PD, and they can require continuous and/or invasive supports, such as ventilation, nasogastric tubes, or percutaneous endoscopic gastrostomy (PEG) [1]. 

The clinical condition associated with infantile PD interferes significantly with children’s quality of life (QoL), that is, the physical, emotional, and social functioning related to their health state [8,9]. Speech/feeding problems or the decline in motor functions limit children’s daily activities, with restricted participation in social and physical activity (mainly due to fatigue in sport practice) [10]. Nevertheless, the early initiation of ERT seems to have a good impact on QoL, facilitating earlier independent walking and/or reducing the need for ventilation [11,12]. Furthermore, learning problems and attention deficits observed in children with PD in comparison with peers, together with negative mood symptoms, may have a negative impact on QoL [13]. However, studies assessing QoL in infantile PD are scarce.

### 1.2. Caring and Parental Burden

Due to the progressive functional impairment in PD, parents undergo significant distress in managing day-to-day living. They live on high alert for their child’s health, particularly for cardiorespiratory crises, and frequently describe their experience as both exhausting and totally involving [14]. The continuous provision of support at home (i.e., managing motor disability/feeding problems) along with ensuring treatments (like ERT twice a month) place an overload of caregiving responsibilities on them. This overload has adverse effects on both the physical and psychological health of parents. In the psychological literature, these consequences are defined as the “burden of care”, a condition experienced by family members caring for a child or relative with a chronic/disabling disease. Caregiver burden encompasses objective changes in the various aspects of life (such as time devoted to care and economic costs) as well as subjective consequences (like emotional strain and familial or social challenges) associated with providing daily care [15,16]. Kanters et al. [17] found higher levels of burden and worse health outcomes among primary caregivers, typically parents (94%), of children with PD compared to caregivers of adults. Higher burden levels were linked to more intense daily care responsibilities, including the time-consuming ERT, the child’s personal care, and social activities. In prioritizing the child’s needs, parents also face difficulties in balancing family life (including marital and sibling relationships) and fulfilling work obligations, with an increase in stress and conflicts [14,18].

### 1.3. The Humanistic Burden

However, the caregiver’s experience is often studded with emotions, worries, and unexpressed needs that extend beyond the negative impact of the clinical and practical consequences of diseases. In a review by Schoser et al. [19], the term “humanistic burden” was introduced to encapsulate the overall implications of PD in both the patient’s and the caregiver’s adjustment. This emphasizes the necessity for a comprehensive understanding of the emotional strain, suffering, and relational and existential needs arising from a rare and disabling disease. However, the authors noted a critical gap in the literature, stating “complete absence of data on the humanistic burden of the IOPD” ([19] p. 12), highlighting a significant void in the literature. Consequently, this study seeks to delve into the unexpressed psychological needs and challenges that contribute to the burden on parents caring for a child with PD.

#### 1.3.1. Living in Uncertainty

A study by Pruniski et al. [20] delved into the psychological consequences experienced by parents who received an early diagnosis of PD following newborn screening (NBS). All parents reported heightened anxiety and fears about the future coupled with a sense of living with uncertainty. Three primary sources of uncertainty emerged: uncertainty about the diagnosis (such as the differences between IPOD and LOPD or the hope for a false-positive NBS), uncertainty about effective treatment (“who to contact? When to start it?”), and concerns about the future, encompassing both the child’s development and family dynamics. Notably, the lack of knowledge about and resources to cope with their baby’s diagnosis was associated with greater anxiety and fear, particularly in cases of early LOPD diagnosis. LOPD is experienced as a “waiting condition”, marked by hypervigilance of the child’s symptoms, an intensified search for assistance (i.e., increased medical consultations or hospitalizations), and worse psychosocial consequences for parents (including emotional strain, cessation of work, and/or financial burden).

#### 1.3.2. The Unspoken Fears

Early identification and ERT have significantly enhanced the prognosis of IOPD. However, despite the treatment, the mortality rate of children from respiratory and cardiac failure remains high [21,22]. The apprehension of sudden death stands out as one of the biggest sources of suffering that parents experience, compounded by the necessity to talk about the child’s illness with the child’s siblings [12].

In numerous instances, parents became “experts” on the rare condition [14,23]. Interestingly, they often exhibit a level of awareness and knowledge surpassing that of professionals (e.g., pediatricians and rehabilitation therapists) regarding recent research or treatments. This reversed parent–professional role may be associated with an increased sense of responsibility in parents. This heightened responsibility manifests in decisions related to treatments and in the exhausting pursuit (“odyssey”) of specialized centers offering more advanced therapeutic interventions [11,23].

Moreover, given that PD is genetically inherited, parents harbor concerns about the potential transmission of the disorder to their other children. This concern can lead to guilt in one of the parents, who may perceive themselves as the cause of the condition [14]. The siblings, in turn, may feel anxious, fearing the onset of similar symptoms. They often experience a sense of isolation and neglect as the illness demands the involvement of their parents, thereby causing siblings to shoulder added responsibilities within the family [24]. Families confronting rare genetic disorders encounter numerous challenges that comprise a condition of burden and uncertainty due to the management of medical complexities, grief, and worries regarding present and future generations [11,25]. 

### 1.4. Protective Factors and Resilience

Research has investigated the protective factors that enhance parents’ adjustment to a rare condition, facilitating the development of positive aspects (such as personal growth, a sense of coherence, and appreciation of life), even while experiencing suffering and the burden of care [14]. Focusing on daily tasks, actively seeking support, and acknowledging the positive aspects of caring for a child with a rare disease were identified as coping strategies linked to a reduced parental burden [26]. Furthermore, parents who possess flexibility, optimism, inner strength and self-efficacy, acceptance, and family cohesiveness are more resilient; they experience better adaptation outcomes and meanings despite their child’s health problems [20,23]. 

### 1.5. Aim and Hypothesis

The current study has two scopes: (a) assessing the health-related QoL in children with diagnosed PD, and (b) delving into the experiences and burdens encountered by parents caring for a child with this rare and severe condition. This study’s hypotheses are outlined as follows.

Firstly, a negative correlation between children’s QoL levels and ages is expected. In fact, despite the observed developmental variability in cognitive, behavioral, and social functioning in children (dependent on factors such as residual enzyme activity and treatment [1,13]), the progression of PD symptoms is well established.

Secondly, the clinical conditions and daily responsibility of care are distressing for parents. Consequently, the hypothesis is that lower QoL levels in children are associated with a higher caregiver burden. 

Thirdly, in accord with van der Borne and colleagues’ model [27,28] on psychosocial problems influencing distress in parents of children with a chronic or life-threatening illness, this study hypothesizes that uncertainty regarding disease/resources and concerns about the child’s future are the primary sources of caregiving burden levels.

Fourthly, this study considers that resilience, defined as positive successful adaptation despite suffering or challenging circumstances [29], has a protective factor for parents facing the demands of their child’s care. Therefore, the hypothesis to be tested is whether greater parental resilience correlates with lower levels of burden. 

Finally, recognizing that parental psychological needs often go unexpressed in questionnaire-based data, this study incorporates semistructured interviews. The interviews were conducted with three volunteer mothers with the aim of exploring the following: (1) What experiences and humanistic burdens do the parents of PD share? (2) How should the social support/interventions be enhanced to prevent parental burden?

## 2. Materials and Methods

### 2.1. Participants and Eligibility Criteria

This study included parents of children and adolescents with a confirmed diagnosis of PD. The inclusion criteria were as follows: being a caregiver of a child (1) up to 18 years old, (2) who had a confirmed diagnosis of IOPD or LOPD, and (3) presenting complex healthcare needs. Given that data were gathered through a self-report questionnaire, foreign parents self-assessed their proficiency in the Italian language, responding affirmatively to a specific question indicating their ability to read Italian.

#### Recruitment

Participants were recruited through invitations to voluntarily engage in an online survey (see section below). The final sample consisted of 12 mothers (M age = 43.2 years, ranging 34 to 52) and 2 fathers (M age = 50.5 years, ranging 44 to 57). Geographically, the families originated from the Northern (14.3%), Central (35.7%), and Southern (50.0%) regions of Italy, with two mothers being of foreign nationality. In term of employment status, the majority of parents were employed (60%), and the educational background was predominantly medium to high (60% high school and 40% degree).

### 2.2. Development of the Survey

The online survey was disseminated in collaboration with the Italian Association for Glycogen Storage Disease (Associazione Italiana Glicogenosi, AIG) and the Rare Disease Observatory (Osservatorio per le Malattie Rare, OMaR). An invitation to participate in this study, providing a brief overview of its aim and procedure, was posted on the Facebook page of AIG (https://www.aiglico.it/la-nostra-storia/; accessed on 14 April 2022) and in OMaR’s online newspaper (https://www.osservatoriomalattierare.it/; accessed on 14 April 2022). Caregivers interested in this study filled out the informed consent and subsequently received the link to fill out the questionnaires online. The online survey was open for data collection from May to August 2022.

Moreover, telephone interviews were conducted for the qualitative part of this study. An invitation to participate in these interviews was published on the Facebook page of AIG. Parents interested in sharing their experiences in caring for a child with PD directly contacted the interviewer and arranged a video call appointment.

### 2.3. Data Collection and Measures

The quantitative data were gathered through self-report questionnaires disseminated online. The online survey comprised three sections: participant demographics, the child’s clinical data, and the psychological needs and adjustment of parents. 

*Participant’s demographics*: The parent’s age, gender, level of instruction, occupation, and marital status variables were collected. Family-related information included the number of children and whether other siblings or family members were affected by PD.

*Child’s clinical data, interventions, and supports:* The child’s age, gender, age at onset of symptoms, age at PD diagnosis, treatments (e.g., ERT, invasive/noninvasive ventilation, and/or PEG), and supports (e.g., speech/physical therapy) were collected. A specific question inquired whether other children or family members had a diagnosed glycogen storage disease. 

Following the input of these data, the survey proceeded to assess the child’s health-related quality of life (HRQOL) using the *Pediatric Quality of Life Inventory* (PedsQL^TM^ [8]; It. ad. [30]). The parent proxy-report questionnaire, a standardized brief scale (23 items), measures core health dimensions outlined by the World Health Organization (physical, social, emotional) and school functioning. For this study, the PedsQL^TM^ 4.0 Generic Core Scales were utilized. Parents selected the child’s age, and the corresponding version of the questionnaire (toddlers 2–4, young children 5–7, children 8–12, and teens 13–18 years old) was made available for online completion. Parents indicated the extent to which a described item had been a problem for the child in the past one month using a 5-point Likert scale (0 = *never a problem* to 4 = *almost always a problem*). Scores were transformed to a 0–100 scale [9], and the Total Scale Score resulted from the sum of all items, with higher scores indicating better HRQOL. The reliability of the HRQOL total scale is excellent (Cronbach’s alpha 0.92 for parent report).

The section on the parent’s *psychological needs and adjustment* involved participants filling out the following self-report questionnaires.

N-DRI, *psychological Needs and Disease-Related Issues*, consists of items that assess the caregiver’s psychosocial needs and strengths in coping with their child’s illness, following the domains proposed by van Der Borne et al. [27]. For the current study, the selected domains from the Italian version of the questionnaire [31] include the following: *Uncertainty about disease* (11 items; e.g., “Quite a lot/very much need of information about: How my child can develop”) and *resources* (8 items; e.g., “How I can talk to or deal with the doctor”); *Fears for the child* (4 items; e.g., “Quite/very much concerned about: Disappointments my child has to cope with the future”); and *Fears for themselves* (10 items; e.g., “Quite/very much concerned about: Coming to the end of my patience in assisting my child”). Additionally, the *Fears for siblings* domain measures worry about concerns for other children (4 items, “Other children will feel neglected by me due to the attention I give to this sick child”) and was included in the Italian version of the questionnaire. Moreover, a specific item was added to this domain for this study given the inherited nature of PD (“I’m concerned that glycogen storage disease could be passed on to siblings as well”). The caregiver responded on a 4-point Likert scale (score ranging from 1 to 4), where higher scores indicated that the parent perceives the disease-related issues as more urgent. The Italian version of the questionnaire demonstrated adequate psychometric characteristics (Cronbach’s alphas ranging from 0.68 to 0.94) in previous studies involving parents of children with life-limiting diseases [28].

CBI—*Caregiver Burden Inventory* [16]. The questionnaire (24 items) evaluates the caregiver’s experience of burden across five dimensions: time dependence (restrictions on daily time), developmental burden (feeling of disappointment regarding one’s aspirations and life projects), physical burden (somatic overload, e.g., chronic fatigue), social burden (conflict with family/job roles), and emotional burden (negative feelings related to the family member requiring care). Each item receives a score ranging from 0 (not at all descriptive) to 4 (very descriptive), with higher scores reflecting a higher perceived burden. The CBI total score categorizes burden conditions into three levels: not problematic (CBI ≤ 24); moderate (24 < CBI < 36), indicating the caregiver’s need for support or replacement in assistance; and severe caregiver burnout (CBI ≥ 36). The reliability of the Italian version of the CBI [32] was excellent (Cronbach’s alpha = 0.95).

The *Resilience Scale for Adults* (RSA) [33] is a brief questionnaire (11 items, RSA-11; It. ad. [34]) designed to evaluate both personal aspects (such as effective coping skills) and contextual resources (including support and family cohesion). Respondents rate each item on a 7-point semantic differential scale, which features a positive/negative attribute at each end of the scale continuum. For instance, an item like “My family is characterised by” presents two opposing responses: “Disconnection” (score 1) and “Healthy cohesion” (score 7). To reduce acquiescence biases, half of the items had a reversed position, with the positive attribute on the left side of the scale. The higher the RSA total score is, the higher the level of the person’s resilience. 

#### Interviews

The interviewer, a psychology trainee with a master’s degree, possessed expertise in selected self-report measures and interview techniques. Before starting, mothers were apprised that the interviews would be recorded, and any confidential information gleaned would be used solely for research purposes, ensuring privacy. Following the receipt of oral informed consent, the interviews commenced with an opening question (*How is your son/daughter?*). Subsequent open-ended questions addressed key topics including the following: (1) diagnosis (e.g., *At what age did you notice the first symptoms? What were they?*); (2) caregiving responsibilities and challenges (e.g., *At what age did you notice the first symptoms? What were they?*); and (3) adaptation and resources (e.g., *Looking back over your story, do you think you can better manage the presence of the disease in your life today?*).

### 2.4. Data Analysis

Quantitative data were processed using IBM SPSS Statistics for Windows 19.0. Demographic characteristics (both caregiver’s and child’s) and descriptive statistics (means and standard deviations) for study measures were computed. Participants were categorized based on their burden levels (not problematic, moderate, and severe) using CBI total scores. Given the small sample size, Spearman’s correlations (rho) were performed to test the associations among the parent’s measures (N-DRI subscales, CBI, and RSA total) and the child’s variables (age and QoL). 

The telephone interviews were transcribed in entirety and underwent the following steps of analysis: (1) Two psychology trainees independently read the mothers’ narrations, identifying core themes. (2) The emerging themes were compared and discussed under the supervision of one of this study’s authors to establish shared thematic categories. Subthemes were also identified and grouped into the major categories (or themes). (3) The mapping of thematic categories was independently utilized by the two trainees for text analysis. (4) The presence of the themes (and subthemes) within the interviews was independently coded by the two trainees (with an agreement ≥ 80) to calculate the frequencies of the themes among the mothers. (5) Finally, text units significant to the parents’ experience were selected and reported in the results section.

## 3. Results

### 3.1. Quantitative Results

#### 3.1.1. Children’s Characteristics

Among the 14 children, 5 were male and 9 were female, spanning ages from 2 to 16 years. One mother had two children with glycogen storage disease (both boys, aged 1 and 4) but responded to the questionnaires for the older child only due to the restriction that the PedsQL^TM^ not to be administered to those under 2 years old. 

Most children (*n* = 8, 57.2%) received their diagnosis within 18 months or a few weeks of life (i.e., 10–40 days; *n* = 3, 21.4%), while the remaining were diagnosed during childhood (2–9 years old; *n* = 3, 21.4%). According to parent-reported data, the onset symptoms encompassed muscle hypotonia, psychomotor development delay, feeding problems, macroglossia, and respiratory difficulties. Some children (*n* = 3) remain asymptomatic, exhibiting the nonclassic infantile-onset form. Almost all children (*n* = 11; 78.6%) undergo ERT twice a month. Due to a more compromised clinical condition, *n* = 3 children (21.4%) rely on PEG for feeding, and *n* = 8 (51.1%) require ventilatory support, with *n* = 3 undergoing invasive ventilation (I–V) through tracheostomy. Additional interventions include physical therapy (*n* = 10, 71%), speech therapy (*n* = 5, 35.7%), and psychological support (*n* = 5, 35.7%). For *n* = 10 children (71%), the QoL levels fell within the clinical range (PedsQL^TM^ total score ≤ 70; see [35]). Table 1 provides a summary of the children’s data.

#### 3.1.2. Parents’ Measures and Correlations

Table 2 summarizes descriptive statistics (*M* and *SD*) of study variables. Parents report higher scores in the domains *Fears for the child* (*M* = 3.41, *SD* = 0.79) followed by *Uncertainty about disease* (*M* = 3.08, *SD* = 0.81) and *Uncertainty about resources* (*M* = 3.02, *SD* = 0.91). According to CBI levels, a not-problematic condition resulted for *n* = 6 (42.9%) mothers and the two participant fathers. Five mothers (35.7%) reported a moderate level of burden, suggesting a need to be replaced or supported in the child’s care, whereas a serious burnout condition was reported by *n* = 3 (21.4%) mothers.

The correlational analysis reveals a strong inverse correlation between the *Caregiver Burden* measure and the QoL index (*p* < 0.01) as well as a strong positive correlation between the *Caregiver Burden* measure and *Fears for themselves* index (*p* < 0.05). Additionally, there is a significant inverse association (*p* < 0.01) between children’s age and QoL scores. While some other relations yielded substantial coefficients (≥0.40), they did not attain statistical significance, likely attributed to the limited sample size (14 or 9 subjects).

### 3.2. Qualitative

Three mothers participated in a telephone interview, with durations ranging from a minimum of 18 to a maximum of 38 min.

*Mother 1* reported that her 13-year-old daughter received a diagnosis of IOPD at 18 days (NBS). The girl has functional impairment, rendering her unable to walk or eat naturally (requiring PEG), and she relies on invasive mechanical ventilation (tracheostomy). ERT is administered twice a month. The girl attends middle school, harbors a passion for football, and enjoys spending free time with her friends.

*Mother 2* shared that her 16-year-old daughter was diagnosed (IOPD) at 40 days when the mother observed the baby’s lack of response to stimuli, such as fixing her gaze. The daughter is undergoing ERT (twice at month), has severe motor disability, relies on PEG for feeding, and requires ventilatory support (tracheostomy). The daughter is devoted to her studies in high school and finds joy in family travel.

*Mother 3* provided information about her 5-year-old child, attending kindergarten, who was diagnosed with a nonclassic form of PD at 1 year. The initial concerns were language and motor delay. The child currently receives ERT twice a month. The disease progression is slow and not disabling, allowing the child to walk, breathe, and feed independently.

The topics emerging from the interviews are summarized in Table 3.

#### 3.2.1. Diagnosis

The disruptive impact of diagnosis, accompanied by emotional reactions ranging from disbelief to discouragement, hopelessness, and anguish, is a common experience. One mother reported relying on spirituality, stating, “*I prayed a lot*”, as a means of overcoming the desperation associated with the prospect of death: “*Certainly, we suffered a lot at the beginning, but at that moment, my expectation was that she would survive, and I had no other expectations*”.

Two mothers also discussed different reactions from their spouses; one remembered the father’s despair that led to a suicide attempt: “*At the beginning, it was a tragedy...My husband attempted suicide. I, on the other hand, experienced a very heavy initial distress, and perhaps I just needed to vent. Once I had released the tension, I asked myself some questions: Do I want the child to be happy? Do I want to do everything to help her survive, or do I want to abandon her? I chose to help her live, and so we made efforts and continue to make efforts to give her the most serene life possible*”.

The development of meaning, as expressed in “*I chose to help her*”, assists the parent in attaining a new equilibrium where the child’s well-being becomes the primary focus. 

#### 3.2.2. Relationships with Healthcare Professionals

The dissatisfaction with healthcare professionals represents a critical issue. The suffering appears to be connected not only to the “odyssey” of multiple consultations (“*I contacted several pediatricians*”) but also to not being taken seriously regarding the symptoms they observed in their infant. Mothers find themselves in a state of confusion and feel overwhelmed by guilt about their situation (“*It was me who wasn’t well, suffering from depression*”).

The delivery of bad news—the unfortunate prognosis—is another painful experience recounted by two mothers: “*When the child had a cardiac arrest, the doctor in the intensive care unit told us that with this condition, reaching one year of age was unlikely, even though they had managed to save her. We also began to have discussions with this doctor because such a thing cannot be said to a parent. You can present them with the situation but stating that the child will die at twelve months, especially when we were already suffering due to the baby’s cardiac arrest and miraculous rescue, was extremely challenging”.*

#### 3.2.3. Responsibilities and Challenges in Caregiving

After the rare disease (referred as the “the monster” by a respondent) entered their family, all mothers expressed the following theme as their daily involvement became exhausting: “*We are all physically and mentally exhausted… It’s not easy at all*”.

Even when the child’s condition is less serious (as mentioned by mother 3), managing daily activities (such as personal hygiene, therapies, and sports and homework) presents challenges. Specifically, two mothers recalled the distress of unsuccessful breastfeeding (mistakenly attributed to the maternal emotional state after diagnosis) and feeding difficulties due to reflux. The mothers were also keenly aware of the relentless progression of PD, particularly concerning motor disabilities: “*Now the situation is deteriorating; my daughter has lost most of her strength and can only move the thumb of her left hand, which she still uses to push her wheelchair, but not always. We must be constantly around her*”. 

Parents navigate a delicate balance between accepting the disease, especially when symptoms remain stable, and experiencing anguish as their child’s condition worsens.

#### 3.2.4. Adaptation

The mothers detailed how the unusual demands of caregiving altered their life: “*I can’t do anything else except for being her mom and her caregiver”*. Parents are compelled to juggle their child’s needs within the family dynamic (e.g., managing siblings), but coping with daily responsibilities necessitates flexibility in family roles: “*I no longer go grocery shopping; my husband does it”*. This adjustment is distressing and often accompanied by a sense of sacrifice. Specifically, the demands associated with medical care take precedence in parents’ lives, inevitably impacting their employment (resulting in two mothers giving up their jobs) and social life. Social isolation, such as seeing friends less or abandoning leisure activities, is a shared experience: “*She consumes a lot of my time, but nothing else gets done (…) There are things I haven’t done in a long time. We only do what is possible with her. We’ve lost some friends, experiences, hobbies, and passions. We’ve become absorbed by this*”.

While prioritizing the child’s needs provides reassurance to the parents, it can strain their marriage, leaving the couple’s needs unmet: “*She is now at peace, but I can’t deny that it has cost us a lot, even as a couple (…) And we should find each other again. It’s been thirteen years since I haven’t slept with my husband; I sleep with my daughter every night. It’s difficult even to find time at night to be together, to spend some time together, or even just to connect. We used to talk about our days in bed, but that doesn’t happen anymore because I sleep with her*”.

#### 3.2.5. Resources

Three protective factors emerged in fostering better adaptation to PD: parental coping mechanisms, social resources, and the child’s resilience. 

##### Coping Strategies

All mothers described normalization during the process of reintegrating the disease into family life: “*Despite the severity, I can still give her baths by the sea, and we manage to do many things. Also, because having to choose between my life and letting M. live, I chose to let her live. She goes to school, studies, and works hard. But she gets tired just as much. In fact, that’s why we study for many hours a day, because she also needs rest”.*

Another mother explained, “*The illness is her life; she doesn’t see it as an illness. She feels unwell when, for example, she has a fever that prevents her from doing what she enjoys, like going to school or playing soccer because the illness is her life, and for us, it’s the same. This new life that has befallen us is our new normal”.*

The discussion about communicating the illness to the child also emerged, with mothers relying on their sensitivity, patience, and realism in providing information: “*I have never lied to her. I’ve always explained everything to her using language suitable for her, because I couldn’t tell her, ‘You will be an unfortunate child.’ I’ve always made her understand that we must try, but we might not succeed, and that in the event of failing, it wouldn’t be a tragedy because there could be other options*”.

In particular, focusing on the present emerged as a common coping strategy: “*When she was little, she wouldn’t eat, so I couldn’t even know if she would walk in the future, but at that moment, I wasn’t thinking about it. It wasn’t the time for her to walk. We were living day by day; we could never be sure if she would make it to the next day, and we adapted to that pace. Then we reached the point where she was supposed to walk, and she didn’t, and… well, it’s okay! We’ll stay in the wheelchair… We’ve been through so much that if she doesn’t walk, it’s okay; the important thing is that she lives*”.

Another mother shared, “*I live day by day, aware of what might happen, but I face things as they come, and in the meantime, I support my child’s development, trying to give him the best life possible, because there might be a time when what he can do now, he may never be able to do again. He can walk now, but it’s not guaranteed that he won’t need assistance in the future. So, I strive to let him fully experience the life he can lead now*”.

When medical supports became vital for the child, even if invasive, they were accepted as “faithful helpers”, and the shock was followed by positive reevaluation. Acceptance is facilitated by mothers recognizing that the child is progressing, so in the mothers’ stories, pain gave way to gratitude and trust: “*The most important thing for us was to save her, so everything else was accepted because the child was alive. The PEG, at ten months, was a significant moment, following which she also had a cardiac arrest (possibly due to anesthesia) [they] also performed stomach closure because she had reflux (when she burped, milk would come back up from her stomach and go into her lungs). It was heartbreaking for us, but it was what could save her, so we accepted it willingly; just this PEG helped her grow. Four months after this operation, they performed a tracheostomy. Until the end, we prayed that we wouldn’t reach that point, and I was desperate at the thought. However, today, I kiss that same tracheostomy every morning… before, she used all her energy to breathe, and she was always tired. Thanks to mechanical ventilation, it was possible to free up that energy and use it for other things: for physical therapy, speech therapy, for sending her to daycare, and now to school*”.

##### Social Resources

The mothers highlighted the advantages of social support from both extended family (e.g., grandparents) and community services: “*When I needed to understand specific aspects of Pompe disease, both psychological support and the medical expertise of the scientific committee were very important to me… It’s reassuring, when I have a problem, I know I’m not alone because the Association can help me. Then we’ve found many people with whom to exchange views and thoughts. Sharing the same condition with others gives you courage*”.

Interestingly, one mother recalled forming a group with other parents of disabled children. Seeking support from others with similar life experience is an effective coping strategy as it reduces feelings of isolation and fosters mutual assistance. Additionally, in the case of rare diseases, linking families with community services (including self-help groups) proves immensely useful for informational support, allowing families access to information about their child’s PD without expending extensive time and financial resources.

##### Resilience of the Child

Finally, recognizing the child’s strength and personal resources helps parents cope with PD, fostering feelings of hope and gratitude. In essence, the resilience of children can serve as a protective factor for parents themselves. Mothers enumerated several positive qualities of their children (e.g., “*intelligent, curious, full of life, tenacious*”), particularly noting that children’s cognitive and school functioning seem to be preserved. Even for the two teenagers facing the most severe PD, motor disabilities or invasive medical supports do not hinder their engagement in a life filled with interests (e.g., sports and theater): “*She’s a child with many hobbies. For example, about a month ago, she found out about a theater show through a YouTube advertisement, and she insisted on going with all her strength. It’s her who gets us out of the house with her interests (…) Day or night, she keeps us on our toes. We’re tired, but so satisfied to have this creature who has been a challenge for everyone*”.

The children’s developmental accomplishments reward the parents’ efforts, and the well-being of the children sustains the family’s hope: “*This arduous journey has rewarded us because we’ve managed to find a balance. She’s a resilient child. Even now, in a wheelchair, with the PEG tube, hearing aids, and glasses because she can’t focus on images, she’s a happy, serene, intelligent child. She has now progressed to the third year of middle school with excellent grades. Hence, this struggle has been worthwhile*”.

#### 3.2.6. Barriers

Mothers expressed their distress regarding the accessibility of community health services. Despite existing public policies for disability, obtaining supports such as medical devices and therapies entails prolonged waits and complex administrative procedures. Mothers openly expressed their anger towards the institutions that fail to uphold their children’s rights: “*It’s not easy at all. Moreover, the institution doesn’t provide much help; you’re constantly chasing after them. The more you depend on the National Health Service (ASL in Italy), the more time you must spend due to the slow bureaucracy*”. 

Additionally, children with PD necessitate coordination among multiple medical specialists and support services (including education at school), thereby placing an increased burden on parents who must liaise with everyone involved. 

Finally, the COVID-19 pandemic was described as an added burden on families. Core concerns revolved around the fear of transmitting the infection and the increased vulnerability of children. Mothers recalled feeling isolated due to physical distancing measures and the escalated responsibilities in caregiving resulting from the suspension of home services: “*With the Covid emergency, all the healthcare professionals were absorbed by vaccination centers and hospitals (…) Also, due to Covid, out of fear of her getting infected, no one has entered our home, not even the girl who used to help her with her schoolwork. I took her place*”.

## 4. Discussion

The main scope of the present study was to expand understanding regarding the psychological impact of rare disorders [14,36] by exploring family experiences with infantile PD. Rare diseases are ranked sixth on the scale of the most stressful existential events [37]. Moreover, in PD, the rarity of the condition is compounded by its progressive neuromuscular consequences, such as motor disabilities, feeding/respiratory deficits, and risk of premature death, all of which significantly impact the child’s QoL and the parental burden. According to Schoser et al. [19], this burden encompasses not only the objective consequences of caring for a child with medical needs, such as daily time for care, but also the emotional, relational, and existential challenges (referred as the “humanistic burden”) that influence parental adjustment to and coping with the disease. Thus, this study aimed primarily to address a gap in the literature, as data on the caregiving burden with children affected by PD are scarce. Secondly, the quantitative assessment of burden was complemented by the qualitative phase of this study to capture the meanings and themes of parental experiences (humanistic burden) with a child having disease-related needs. It is crucial to note that the number of participants was small (*N* = 14 parents, primarily mothers, with only *n* = 3 participating in the interviews), which was expected due to the rarity of PD. However, the current study bridges a gap in the existing literature on the parental experience with a child with PD and provides suggestions for interventions.

### 4.1. Health-Related Quality of Life and Caregiver Burden

Children’s QoL declines with age, and the majority of children (*n* = 10, 71%) exhibit a clinically significant impairment in physical, emotional, and social functioning. Specifically, a deterioration of motor functions over time emerges as a central concern in maternal narratives, aligning with clinical data on long-term outcomes in infantile PD [12]. Furthermore, as hypothesized, a higher caregiver burden (as indicated by CBI scores) correlates with poorer QoL in children. These findings are in line with observations in adults with PD, where a greater burden was linked to a lower QoL (particularly motor disability but not alertness for cardiorespiratory crises [17]). The narratives of mothers corroborate their physical fatigue in daily activities, especially when the child’s motor skills are residual (e.g., a daughter in a wheelchair who can move only one finger). Moreover, the CBI scores suggest a condition of severe burden experienced by mothers (21.4%), with an increasing number (35.5%) expressing a need to be relieved of their daily caregiving responsibilities. Parents might benefit from additional practical support, particularly when the demands of the child’s daily care surpass their individual resources. However, studies, e.g., [14,23], suggest that seeking assistance may be more challenging in the context of rare diseases; parents might fear that others lack knowledge about the child’s special needs (such as managing crises or feeding), leading to reluctance in being replaced in caring. Hence, education for both formal (e.g., nurses or teachers) and informal caregivers is pivotal to ensure supportive service and home care for children with complex health needs. Simultaneously, if parents perceive the provision of skilled care as satisfactory, they may experience relief or respite from their caregiving duties, consequently reducing the risk of burden. 

Parental burden is also associated with fears of negative consequences for themselves, such as loneliness, job loss, or the inability to cope with the weight of disability in the future. The data also confirm well-known gender differences, indicating that fathers experience a lower burden compared with mothers [28]. This primary caregiving role assigned to mothers aligns with the typical family organization in the Italian context [23]. It is imperative that interventions consider this specific vulnerability of mothers, enhancing the coparenting, communication, and emotional support between partners. Additionally, since the quality of the couple’s relationship is impacted, parents should be supported in nurturing their well-being and preserving their marriage. This aspect remains an overlooked area in research, warranting future studies to explore the effectiveness of family-based interventions specifically tailored for these burdened parents.

### 4.2. Coping Strategies

Regarding disease-related concerns, parents express heightened worries about their child’s future, such as worsening of the child’s disability or facing disappointments, compared to other sources of concern, such as uncertainty regarding the disease/resources or concern related to other siblings. The narratives from mothers underscore an awareness of the relentless progression of PD, with a particular focus on the remaining self-management and motor skills as central concerns. However, adopting the normalization of the disease—essentially striving to reconstruct “normality” in the child’s life and within the family—emerges as a coping strategy to alleviate the anguish associated with the future. Specifically, factors such as the stability of symptoms, the child’s functioning at school, or the ability to pursue interests during leisure free time serve as reassuring aspects of the child’s life that alleviate emotional suffering in mothers. These findings contribute to expanding the knowledge regarding the protective role of rare disease normalization within family life [20]. Conversely, when the caregivers perceive an inability to control the “normality” in their lives or in their child’s health, there is an increase in general stress or disease-related burden [38]. 

Positive reappraisal and focusing “on the present” (e.g., daily tasks) emerge as other coping strategies derived from maternal experiences. Positive reappraisal enables individuals to “think differently” about an adverse experience (such as invasive mechanical ventilation or PEG) by reconceptualizing it as beneficial rather than negative (e.g., improving the child’s QoL by reducing respiratory distress through mechanical ventilation). Research confirms that positive reappraisal serves as an adaptive mechanism for regulating negative emotions and is linked to improved parental adjustment to rare diseases [14]. Regarding focusing “on the present”, mothers articulate their efforts to learn from experience and provide their children with “the best possible life” without anticipating future problems (e.g., assuming the child will not walk and will require a wheelchair). Interestingly, mothers draw strength from their children, describing them as “serene, vital, tenacious, and patient”, despite their progressive and life-threatening problems. In maternal narratives, these positive qualities are associated with greater appreciation for life and a greater acceptance of disease and/or disability. Additionally, prior studies documented that the perception of a child’s vulnerability is positively associated with the parent’s stress [39]. Future research could delve into how parents’ perceptions of their children’s qualities may impact their adaptation to PD. 

### 4.3. Benefits of Support and Resilience

The utilization of social support emerges as an external coping strategy that has proven beneficial in family experiences with PD. Interestingly, mothers highlight their efforts to establish a support network (forming a self-help group with parents who share similar life experiences) to navigate a tragic event (such as a father’s suicide attempt). Specifically, social support becomes indispensable in the context of rare diseases, where parents fears that other might lack information about the disease and fail to comprehend the child’s care needs [14,23]. The protective role of contextual support—both formal (e.g., health professionals) and informal (such as the extended family)—was affirmed by the additional challenges families faced during the COVID-19 pandemic [40]. Mothers articulated how the emergency’s restrictive measures abruptly altered home care and halted professional supports, leading to an increased sense of isolation, fears, and burden of caring responsibilities (e.g., mother taking the role of educator for homework). Establishing online support (such as websites dedicated to rare diseases) can serve as a beneficial and cost-effective strategy for parents [36]. Maintaining connections with professionals and other families alleviates the loneliness and distress experienced by parents when they feel more vulnerable. Furthermore, given the rarity of many diseases, online support can facilitate mutual help among parents with similar needs, despite geographical distance and the practical difficulty to meet continuously. While studies indicate that parents tend to appreciate self-help groups as a primary source of support over formal assistance from professionals [23], the empirical literature remains limited. Future research should evaluate the effectiveness of various supportive interventions, including innovative online support, in mitigating feelings of isolation and reducing burden levels in parents. 

Contrary to our initial hypothesis, no association was found between burden levels and parental resilience—the individual’s dynamic capacity to achieve a better adaptation despite adverse life circumstances. It is plausible that the global scale [33] employed in this study might be poorly sensitive to capturing caregiving experiences, but it considers broader family factors (e.g., positive affect, cohesiveness, or perceived support) that positively influence resilience. Conversely, previous studies identified coping style, hope and optimism, self-efficacy, and social support as resilience factors for parent of children with disabilities or neurodevelopmental disorders [41,42]. Further research is warranted to explore resilience factors in the field of rare life-threatening childhood diseases.

In conclusion, several parallels emerged between the recurring issues regarding parental experiences with disability/chronic diseases in children [14,21] and the qualitative themes identified in the current study. The parallels encompass emotional reactions after diagnosis, uncertainties stemming from a lack of information and support, the daily stress and burden, and the waivers concerning work/social life opportunities. Furthermore, two themes assume particular significance from the perspective of mothers, yet they frequently remain unexpressed in quantitative studies: (1) the possibility of the child’s death, and (2) the need for supportive communication from healthcare practitioners.

### 4.4. Bad News and the Need for Supportive Communication

Mothers expressed anguish regarding the potential loss of their children’s lives when the children were in jeopardy. However, any relief experienced after a severe crisis was dampened by doctors’ cold communication about the low likelihood of the child’s survival. Therefore, the role of sensitive and empathetic communication from health professionals is paramount in aiding parents in adjusting to bad news. As emphasized by Fernandes and colleagues [43], empathetic communication of the truth has the potential to empower individuals undergoing treatment. It may “reduce feelings of guilt and false beliefs, besides being an opportunity to discuss preferences, plan goals with shared decision-making, and promote a sense of control and security (…). In contrast, inappropriate conversations may cause a more negative impact than all the struggle and hardship related to continued care” (p. 9/15). Consequently, it is recommended that health professionals receive training in communication and supportive intervention to effectively address the emotional needs of parents. Proficient training should enable professionals to discern parental vulnerability and feelings (such as disbelief, anguish, and anticipatory grief) and understand how these feelings can change over time (for instance, postdiagnosis or following an acute crisis [36]).

The present study has some limitations. Firstly, the number of participating mothers is small, with very limited involvement from fathers, despite recruitment efforts through family associations and national centers for rare diseases. This small size number reduces the validity and generalizability of the findings. However, as observed in similar studies involving rare disease populations [17], the limitation imposed by a small number of observations is compensated for by a notably high response rate. Furthermore, the qualitative data obtained from semistructured interviews complemented the quantitative data, allowing for a deeper exploration of existential aspects that often remain unexplored in self-report questionnaires. Interviews were particularly valued by the participating mothers. Secondly, the interviewer has a family member affected by PD. While this circumstance allowed for an empathetic approach, it could potentially lead to a bias during the interview, despite the structured nature of the questions. Thirdly, this study is web-based; therefore, clinical data were solely collected from parental reports, such as the age of symptom onset. A bias due to inaccurate information or false memories cannot be ruled out. Additionally, due to the young age of some children, the assessment of QoL was parent-reported and employed a generic health-related measure. Future studies could benefit from employing a multimethod assessment of QoL, incorporating both proxy measures and the child’s perspective, particularly in assessing difficulties across significant life domains (such as physical, emotional, social, and school functioning). A longitudinal design approach could also be considered for future studies to explore the developmental trajectories of children/adolescents with PD and how the domains of QoL change following intervention (like ERT treatment or mechanical ventilation). 

Despite these limitations, the current study covers the scarcity of empirical data on complex care needs with children suffering from PD. It provides insight for supportive interventions and strategies aimed at preventing the burden of care in parents.

## 5. Conclusions

This study indicates that 57.1% of parents with children affected by PD experience a moderate or severe burden. This burden encompasses not only the challenges associated with daily caregiving but also extends to what is termed as “humanistic burden”, which necessitates interventions integrating medical care with psychosocial support and education. Recently, Fernandes et al. [43] proposed guidelines for providing emotional support while communicating the diagnosis of a neuromuscular disease. The outlined steps for empathetic communication with family/patients include the following: (1) addressing doubts regarding complications; (2) alleviating fear and anxiety; (3) involving the patients and their family in decision making, when feasible; (4) demonstrating compassion and acknowledging spiritual needs; and (5) seeking spiritual support to find meaning in illness, suffering, and death.

These steps align with an integrated, multiprofessional approach to pediatric palliative care (PPC), defined as “the active total care of the child’s body, mind, and spirit”. This approach not only encompasses comprehensive support for the family but also extends its focus to the healthcare team, as detailed in the World Health Organization’s fact sheet on palliative care for children (see https://www.who.int/europe/news-room/fact-sheets/item/palliative-care-for-children; accessed on 30 October 2023).

The PPC approach encompasses several key aspects: (1) preparing parents to receive and educate physicians on effectively communicating an unfortunate diagnosis; (2) assessing both the children’s needs and the psychological requirements of parents, including disease-related issues across various psychosocial domains as defined by [27]; (3) establishing and executing a multiprofessional intervention plan, collaboratively developed by healthcare teams in conjunction with parents and the young patient; and (4) scheduling regular supervision and intervision sessions among healthcare team professionals.

Rare diseases, particularly PD, significantly and detrimentally affect various aspects of daily family life. An integrated PPC approach aims to addresses all identified needs, encompassing both medical and psychosocial dimensions (i.e., emotional, developmental, relational, and educational needs).

Certain recommendations are essential (for a comprehensive review, refer to [18]). For children, attention should be directed towards intersecting identities and experiences, behavioral health, and communication. Regarding families, focus areas include the social determinants of health, such as financial challenges; the feasibility of chronic home health services if required; accessing comprehensive information about disease characteristics and prognosis; and overcoming barriers to well-being, like isolation and loneliness. Importantly, families should be integrated into the care team as they constitute an expert system in managing the child’s and their own daily life, encompassing their preferences, tendencies, habits, and more.

The aforementioned recommendations necessitate a dual-level response, both at an individual professional level and at an institutional level within healthcare services. Italy exhibits exemplary instances of this dual-level approach, such as public services where multiprofessional teams consisting of pediatricians, physiatrists, psychologists, pediatric nurses, physiotherapists, social workers, spiritual assistants, volunteers, and others provide comprehensive care for PPC-eligible children and their families. Italy’s national and regional laws ensure universal free care, granting every individual the right to access PPC and pain therapy, addressing financial hardships as noted by [18].

At the individual professional level, multiprofessional team structures cater to the specific needs of pediatric patients and their families, encompassing hospital, hospice, and home-based treatments. These initiatives include educational sessions tailored to both staff and family members; see [44,45,46,47]. Hence, expanding these PPC methodologies, with a targeted focus on identified needs, represents a unique approach to bolstering social support. This approach serves as a means to enhance parents’ coping strategies and prevent their burden.

## Figures and Tables

**Table 1:** Children’s clinical characteristics (*N* = 14), therapies/supports, and quality-of-life levels.

Age (Years)	Sex	Age at Diagnosis ^1^	ERT ^2^	Ventilation ^3^	Nutrition Support	Physical Therapy	Speech Therapy	Psychological Support	Quality of Life ^4^
4	M	1 y	Y	N	N	Y	Y	N	73.81
4	F	10 d	Y	N	N	N	N	N	64.29
2	F	2 m	N	N	N	N	N	N	96.88
5	M	1 y	Y	N	N	Y	Y	N	51.04
7	M	3 m	Y	IV	PEG	Y	Y	N	29.17
10	F	5 y	N	N	N	N	N	N	82.61
9	F	3 y	N	N	N	N	N	N	69.57
10	F	11 m	Y	N-IV	N	Y	N	Y	43.48
10	F	14 m	Y	N-IV	N	Y	Y	Y	45.65
10	F	14 m	Y	N-IV	N	Y	Y	Y	52.17
13	M	2 y	Y	N-IV	N	Y	N	Y	43.48
13	M	15 m	Y	N-IV	N	Y	N	Y	56.52
13	F	18 d	Y	IV	PEG	Y	N	N	47.83
16	F	40 d	Y	IV	PEG	Y	N	N	71.74

Note: ^1^ d = days, m = months, y = year(s); ^2^ ERT = enzyme replacement therapy, N = no, Y = yes; ^3^ Ventilation: N = no ventilation support, N-IV = yes, noninvasive ventilation, IV = yes, invasive ventilation; PEG = percutaneous endoscopic gastrostomy; ^4^ PedsQL^TM^ total score.

**Table 2:** Study variables’ descriptive statistics (*M* and *SD*) and related Spearman’s correlation coefficients (*r_s_*; *N* = 14).

Measures		1.	2.	3.	4.	5.	6	7.	8.	9.
	*M*	7.93	59.16	3.08	3.02	3.41	1.80	2.21	26.07	56.79
	*DS*	4.10	18.19	0.81	0.91	0.70	0.81	0.79	13.02	12.61
Child’s age	*r_s_*	1								
2. Quality of life	*r_s_*	−0.57 **	1							
3. Uncertainty about disease ^a^	*r_s_*	−0.27	0.37	1						
4. Uncertainty about resources ^a^	*r_s_*	−0.10	0.26	0.85 ***	1					
5. Fears for the child ^a^	*r_s_*	−0.17	0.18	0.58 **	0.58 **	1				
6. Fears for siblings ^a^ *	*r_s_*	−0.40	−0.10	0.21	0.22	0.23	1			
7. Fears for themselves ^a^	*r_s_*	0.44	−0.51	0.24	0.25	0.39	0.08	1		
8. Caregiver burden ^b^	*r_s_*	0.28	−0.67 ***	−0.26	−0.27	−0.14	0.47	0.57 **	1	
9. Resilience ^c^	*r_s_*	0.05	−0.28	−0.28	−0.08	−0.45	−0.29	−0.28	0.01	1

Note: Quality of life = PedsQL^TM^; ^a^ *psychological Needs and Disease-Related Issues* (N-DRI) subscales; ^b^ *Caregiver Burden Inventory*; ^c^ *Resilience Scale*. * *N* = 9; ** *p* < 0.05, two-ties; *** *p* < 0.01, two-ties.

**Table 3:** Themes and subthemes from the analysis of the interviews.

Theme	Subtheme	Frequency	Interview Excerpts
Diagnosis	Dissatisfaction with physicians	Mother 1 Mother 2	*When I realized that my daughter was not feeling well, I contacted several pediatricians. However, the conclusion was always the same: it was me who was not doing well, suffering from depression, and in need of psychological support.*
	Emotional reactions (mother)	Mother 1 Mother 2	*At first, I was desperate.*
		Mother 1 Mother 2	*I was angry, why me?*
		Mother 1	*I prayed a lot.*
	Emotional reactions (father)	Mother 1	*My husband didn’t react well to the diagnosis; the strong one is me.*
		Mother 2	*My husband attempted suicide.*
	Inauspicious prognosis	Mother 1 Mother 2	*She won’t make it to one year of age.*
Responsibilities and challenges in caregiving	Early caring	Mother 1	*I was primarily feeling anger for not being able to breastfeed, maybe because she wasn’t drawing milk, or maybe because I didn’t have any due to the upheaval following the diagnosis (…) It lasted for a month, then I stopped because it was too stressful.*
	Distress	Mother 1	*We should never let go, but we are also very tired because all the various steps require effort.*
Adaptation	Couple’s relationship	Mother 1	*We should find time for ourselves (…). Before, we used to talk about our day in bed, but it doesn’t happen anymore because I sleep with her.*
	Work	Mother 2 Mother 3	*I had to leave my job because the therapy infusions take up a lot of time.*
	Social life	Mother 1 Mother 2	*We lost a part of friends, experiences, hobbies, passions. We have erased ourselves.*
Protective factors	Coping strategies	Mother 1 Mother 2 Mother 3	*I know what could happen, but I deal with things as they come. I’m committed to letting him live the fullest life he can now. As long as the child is well, we fight, without thinking about the drama.*
		Mother 1	*I kiss that ‘little thing’ (tracheostomy) every day.*
		Mother 1	*In my town, there was nothing for disabilities, so five moms and I created this association, and later on, other parents joined.*
	Social support	Mother 1 Mother 3	*The association was essential for me.*
		Mother 2	*We are fortunate to have all four grandparents*.
	Resilience of the child	Mother 1 Mother 2 Mother 3	*Together, we can do many things. She has grown up with the belief that she can do everything with calm and patience.*
			*She is aware of her limits.*
			*Even now, being in a wheelchair with a PEG tube, hearing aids, and glasses because she can’t focus on images, she is a happy, serene, and intelligent child.*
Barriers	Institutions and bureaucracies	Mother 1 Mother 2 Mother 3	*You have to waste time due to the slow bureaucracy.*
	COVID-19 pandemic	Mother 1 Mother 2 Mother 3	*Out of fear that she might get infected, no one ever came into the house, not even the girl who used to help her with her homework. I took her place.*

## Data Availability

The dataset analyzed in this study can be requested from the first author (loredana.benedetto@unime.it.) upon reasonable request.
